# *DLG5* variants are associated with multiple congenital anomalies including ciliopathy phenotypes

**DOI:** 10.1136/jmedgenet-2019-106805

**Published:** 2020-07-06

**Authors:** Jonathan Marquez, Nina Mann, Kathya Arana, Engin Deniz, Weizhen Ji, Monica Konstantino, Emily K Mis, Charu Deshpande, Lauren Jeffries, Julie McGlynn, Hannah Hugo, Eugen Widmeier, Martin Konrad, Velibor Tasic, Raffaella Morotti, Julia Baptista, Sian Ellard, Saquib Ali Lakhani, Friedhelm Hildebrandt, Mustafa K Khokha

**Affiliations:** 1 Pediatric Genomics Discovery Program, Department of Pediatrics and Genetics, Yale University School of Medicine, New Haven, Connecticut, USA; 2 Division of Nephrology, Boston Children's Hospital and Harvard Medical School, Boston, Massachusetts, USA; 3 Pediatric Genomics Discovery Program, Department of Pediatrics, Yale University School of Medicine, New Haven, Connecticut, USA; 4 Department of Clinical Genetics, Guy's Hospital, London, UK; 5 Department of Obstetrics, Gynecology, and Reproductive Sciences, Yale University School of Medicine, New Haven, Connecticut, USA; 6 Department of General Pediatrics, University Hospital Münster, Münster, Germany; 7 Department of Pediatric Nephrology, University Children's Hospital, Skopje, North Macedonia; 8 Department of Pathology, Yale University School of Medicine, New Haven, Connecticut, USA; 9 Exeter Genomics Laboratory, Royal Devon & Exeter NHS Foundation Trust, Exeter, UK; 10 Institute of Biomedical & Clinical Science, College of Medicine and Health, Exeter, UK

**Keywords:** genetics, hydrocephalus, molecular genetics, renal medicine, developmental

## Abstract

**Background:**

Cilia are dynamic cellular extensions that generate and sense signals to orchestrate proper development and tissue homeostasis. They rely on the underlying polarisation of cells to participate in signalling. Cilia dysfunction is a well-known cause of several diseases that affect multiple organ systems including the kidneys, brain, heart, respiratory tract, skeleton and retina.

**Methods:**

Among individuals from four unrelated families, we identified variants in *discs large 5* (*DLG5)* that manifested in a variety of pathologies. In our proband, we also examined patient tissues. We depleted *dlg5* in *Xenopus tropicalis* frog embryos to generate a loss-of-function model. Finally, we tested the pathogenicity of *DLG5* patient variants through rescue experiments in the frog model.

**Results:**

Patients with variants of *DLG5* were found to have a variety of phenotypes including cystic kidneys, nephrotic syndrome, hydrocephalus, limb abnormalities, congenital heart disease and craniofacial malformations. We also observed a loss of cilia in cystic kidney tissue of our proband. Knockdown of *dlg5* in *Xenopus* embryos recapitulated many of these phenotypes and resulted in a loss of cilia in multiple tissues. Unlike introduction of wildtype *DLG5* in frog embryos depleted of *dlg5,* introduction of *DLG5* patient variants was largely ineffective in restoring proper ciliation and tissue morphology in the kidney and brain suggesting that the variants were indeed detrimental to function.

**Conclusion:**

These findings in both patient tissues and *Xenopus* shed light on how mutations in *DLG5* may lead to tissue-specific manifestations of disease. DLG5 is essential for cilia and many of the patient phenotypes are in the ciliopathy spectrum.

## Introduction

Ciliopathies lead to a broad range of disease phenotypes due to abnormalities of cilia assembly, disassembly, motility and/or function.^1–5^ Cilia play diverse roles, from driving extracellular fluid flow to cell-cell signalling, depending on the protein composition of the cilium that can vary between cilia types across tissues.^6^ Thus, ciliary dysfunction may differentially affect a variety of tissues including the brain, retina, heart, respiratory tract, skeleton, kidneys and urogenital tract.^7–14^ As a consequence, dysfunction of different cilia-related genes can cause remarkably different phenotypes across different tissues. Although several monogenic causes of ciliopathies have been identified,^15^ different alleles of the same gene can also cause strikingly diverse disease phenotypes. For example, *CEP290* variants cause phenotypes including Bardet‐Biedl syndrome, Joubert syndrome, Meckel‐Grüber syndrome, nephronophthisis and Senior‐Loken syndrome, each with different as well as overlapping phenotypes.^16^ Finally, patients with identical alleles can also have distinct phenotypes that are difficult to explain mechanistically, reflecting our still poor understanding of cilia function and the underlying human genetics.^17 18^

Here, in a cohort of four unrelated families, we describe an association of *discs large 5* (*DLG5*) with multiple different patient phenotypes including those associated with ciliopathies. Dlg5 is a member of the membrane-associated guanylate kinase family of proteins and participates in regulation of Hippo, sonic Hedgehog (Shh) and transforming growth factor beta (TGF-β) signalling.^19–21^ Dlg5 also maintains cell polarity through interactions with β-catenin and the vinexin-vinculin complex.^22–24^ Interestingly, the patient phenotypes are variable, each presenting with a different constellation of findings, including congenital anomalies of the kidney and urinary tract (CAKUT), steroid-resistant nephrotic syndrome (SRNS), congenital heart disease (CHD), skeletal abnormalities and hydrocephalus. Initially, a commonality across patients was not obvious. However, assembly of the full cohort of patients, coupled with functional testing in our high-throughput human disease model, *Xenopus*, revealed a ciliopathy phenotype that we observed in one patient’s tissues, demonstrating a loss of cilia in a tissue-specific manner. Interestingly, the patients had different alleles and modes of inheritance with *de novo*, heterozygous, homozygous and compound heterozygous variants. Exploiting our animal model, we tested these individual patient variants and demonstrated a loss of function for each. Our work establishes *DLG5* as a ciliopathy gene that can present with a variety of different phenotypes.

## Methods

### Clinical proband samples

We collected blood samples and pedigree information after informed consent from individuals or their guardians.

### Whole exome sequencing

Whole exome sequencing was performed on genomic DNA isolated from blood lymphocytes or saliva and subjected to exome capture using IDT xGen human exome capture arrays (Integrated DNA technologies) followed by next-generation sequencing on a HiSeq4000 Illumina sequencing platform. Paired end sequence reads were mapped to the human reference genome (NCBI build 37/hg19), and the variants were called using GATK and annotated using AnnoVar.^25^ Variants with minor allele frequencies >1% in any subpopulation from the gnomAD v2 database were excluded. Remaining variants were evaluated and ranked based on established criteria^26–28^ and pedigree information. Variants were confirmed via Sanger sequencing. Variant nomenclature was based on NM_004747.3.

### *Xenopus* embryonic manipulations

*Xenopus tropicalis* were housed and cared for according to established protocols approved by Yale IACUC. Using standard protocols, we injected MOs (10 ng into one cell stage embryos or 5 ng into one cell of a 2 cell stage embryo), fluorescent tracer (Mini-ruby) and/or mRNA into one cell stage (100 pg) or one cell of a two-cell stage (50 pg) *Xenopus* embryos. CRISPR/Cas9-mediated genome editing in *X. tropicalis* embryos was used as previously described.^29^ A CRISPR sgRNA targeting exon 1 of *dlg5* was designed to generate F0 knockout tissue in embryos (GGTAAAGGATGGAGAGGAGG). For loss-of-function experiments, 400 pg sgRNA along with 1.2 ng Cas9 (CP03, PNA Bio) in a 2 nL volume were injected into one-cell stage embryos. Alternatively, for targeted loss-of-function experiments 200 pg sgRNA along with 0.6 ng Cas9 (CP03, PNA Bio) in a 2 nL volume were injected into one cell of two-cell stage embryos while the other cell was injected with 0.6 ng Cas9 (CP03, PNA Bio) in a 2 nL volume. We generated *in vitro* capped mRNA of wildtype and variant human *DLG5* from sequences (BC146794) cloned into the pBluescript II SK+backbone using the T7 mMessage machine kit (Thermo Fisher) following the manufacturer’s instructions followed by polyadenylation using *Escherichia coli* poly(A) polymerase (NEB). The transgenic line *Xtr.Tg(pax6:GFP;cryga:RFP;actc1:RFP)^Papal^* that was used to visualise Pax6 expression changes was obtained from the National Xenopus Resource. 30

### Whole mount in situ hybridisation

Whole mount in situ hybridisation (WISH) was carried out as previously described.^31^ Briefly, *Xenopus* embryos were fixed in 4% paraformaldehyde and dehydrated through washes in methanol. Embryos were rehydrated in phosphate-buffered saline (PBS) with 0.1% Tween-20. Embryos were then hybridised with digoxigenin-labelled antisense RNA probes complementary to *dlg5, dnah9, foxa1 or ptch1* generated using the T7 High Yield RNA Synthesis kit (NEB, E2040S) and DIG-dUTP (Sigma). Embryos were then washed and blocked prior to incubation with anti-DIG-Fab fragments (Roche) overnight at 4°C. BM purple (Sigma) was used to visualise expression prior to postfixation in 4% paraformaldehyde with 0.1% glutaraldehyde.

### Optical coherence tomography imaging/measurements of brain ventricles

Stage 45 *Xenopus* embryos were immobilised and imaged using a Thorlabs Telesto 1325 nm spectral domain-optical coherence tomography (OCT) system as previously described.^32^ The brain two-dimensional cross-sectional images and movies were obtained and measured using the integrated Thorlabs software.

### Immunofluorescence and microscopy

Both *Xenopus* and human tissue samples were first briefly permeabilised in PBS with 0.1% Tween-20 followed by fixation in 4% paraformaldehyde. For pronephric tubule imaging, *Xenopus* tails and ventral tissue were dissected away prior to clearing in Sca*l*eS4 for 5 days and incubating with immunohistochemical labelling reagents overnight.^33^ Proximal tubules were identified through labelling with fluorescein-conjugated lectin. Immunofluorescence reagents are listed in online supplementary table 1. Tissues and embryos were imaged on a Zeiss LSM880 confocal microscope.

10.1136/jmedgenet-2019-106805.supp3 Supplementary data

### Image analysis

All fluorescent micrographs as well as WISH images were processed and analysed using Fiji.^34^ To limit quantitation to the same tissues between control and *dlg5* depleted pronephroi, we restricted our investigation to only the proximal tubules labelled with fluorescein-conjugated lectin. Proximal pronephric tubules were evaluated for both maximal diameter as well as cilia number along 500 nm cross-sections including the tubule portion with the widest diameter as determined by a z-stack obtained during imaging. Maximal diameter was established within the lumenal space on the micrographs of phalloidin signal which delineated the cell borders within the tubule. Cilia number quantitation was aided by using the skeletonize function and count particles function in FIJI on the micrographs of antiacetylated α tubulin signal. A workflow of this approach is depicted in online supplementary figure 1.

Density quantitation of multiciliated and goblet cells in the *Xenopus* embryonic epidermis was carried out by manually designating cells as a multiciliated cell based on positive antiacetylated α tubulin signal or goblet cell based on lectin signal over a constant imaging area.

### Statistical analyses

All *Xenopus* experiments were performed a minimum of three times and numbers stated in graphs are the composite of multiple experiments. Statistical significance of abnormalities was tested using two-tailed t-tests or analysis of variance followed by Dunnett’s multiple comparison tests. In all figures, statistical significance was defined as p<0.05.

## Results

### Variants in *DLG5* cause a constellation of congenital anomalies

**Figure 1:**
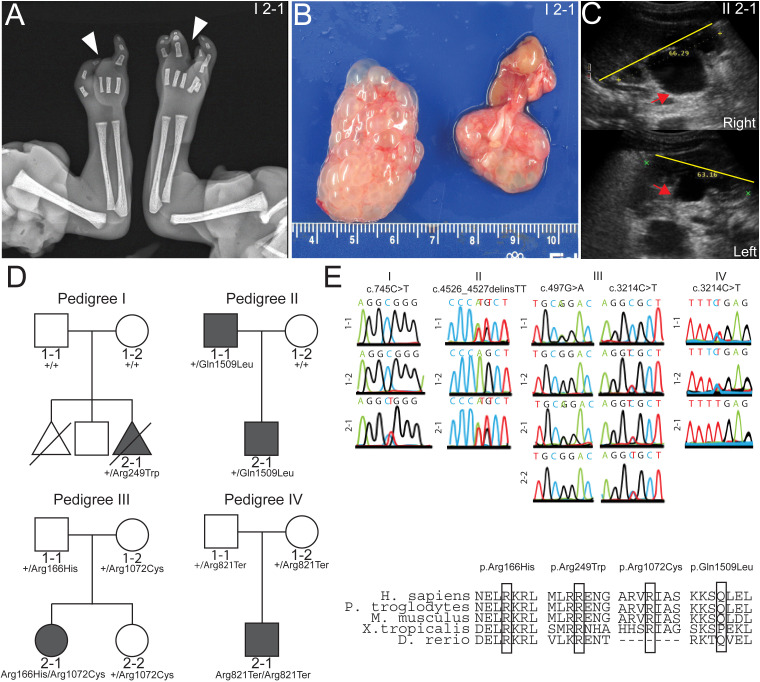
Whole exome sequencing identifies *discs large 5* (*DLG5)* variants in patients. (A) Radiograph of patient I 2–1 fetal upper extremities reveals bilateral ectrodactyly. (B) I 2–1 fetal kidneys were largely cystic and dysplastic. (C) Ultrasound of II 2–1 kidneys show hydronephrosis. Yellow line depicts span of kidney and red arrows indicate dilated renal pelvis. (D) Pedigrees depict families in which the *DLG5* variants were identified. (E) Sanger sequencing confirming variant allele presence and amino conservation through phylogeny for the mutated allele of *DLG5*.

The proband (I 2–1) was a 21-week gestational age male fetus with skeletal abnormalities including bilateral ectrodactyly (figure 1A) and bilateral clubfeet. The fetus also had CHD with a membranous ventricular septal defect and overriding aorta. Finally, the fetus had bilateral multicystic dysplastic kidneys (figure 1B) and oligohydramnios. To identify potential pathogenic variants, we performed whole exome sequencing of the proband (I 2–1) and the non-consanguineous healthy parents (I 1–1 and I 1–2) (figure 1D). Sequencing analysis identified a putatively damaging *de novo* variant (c.745C>T, p.Arg249Trp) in the gene *DLG5*, confirmed by Sanger sequencing (figure 1E). No variants were identified for known causes of CAKUT in this patient. This variant occurred at a CpG site within exon 5 of *DLG5* and has an allele frequency of 1.23e-5 with no homozygous individuals in the gnomAD database (table 1). This non-conservative missense variant had CADD score of 27 and was predicted to be deleterious by multiple lines of computational evidence (CADD, SIFT, Polyphen). The residue Arg249 is evolutionarily conserved among multiple species (table 1).

**Table 1:** Mutations in *DLG5* in five individuals from four families with congenital anomalies and/or SRNS

Family (individual)	Phenotype	Nucleotide change	Amino acid change	Exon (zygosity)	PPH2 score	CADD score	SIFT	MT	Species of amino acid conservation	gnomAD	Sex	Ethnic origin
III 2–1	SRNS	c.497G>A	p.Arg166His	Exon 3 (Cmp)	0.956	23.6	Del	DC	Pt, Mm, Xt, Dr	0/5/245918	Female	European
I 2–1	CAKUT, CHD, LA	c.745C>T	p.Arg249Trp	Exon 5 (DN)	1	27	Del	DC	Pt, Mm, Xt, Dr	0/3/242960	Male	European
IV 2–1	CFD, HC, LA	c.2461C>T	p.Arg821Ter	Exon 15 (Hom)	NA	40	NA	NA	NA	NP	Male	European
III 2–1	SRNS	c.3214C>T	p.Arg1072Cys	Exon 15 (Cmp)	0.818	29.9	Del	DC	Pt, Mm, Dr	0/154/277032	Female	European
II 1–1	CAKUT	c.4526AG>TT	p.Gln1509Leu	Exon 24 (Het)	0.179	23.6	Del	DC	Pt, Mm, Dr	NP	Male	European
II 2–1	CAKUT	c.4526AG>TT	p.Gln1509Leu	Exon 24 (Het)	0.179	23.6	Del	DC	Pt, Mm, Dr	NP	Male	European

http://gnomad.broadinstitute.org

CADD, combined annotation-dependent depletion; CAKUT, congenital anomalies of the kidney and urinary tract; CFD, craniofacial defects; DC, disease causing; *DLG5*, *discs large 5*; Dr, *Danio rerio*; gnomAD, Genome Aggregation Database; HC, hydrocephalus; Het, heterozygous; Hom, homozygous; LA, limb abnormalities; Mm, mus musculus; MT, Mutation Taster (prediction software); NA, not applicable; NP, not present; PPH2, PolyPhen-2 (prediction software); Pt, pan troglodytes; SIFT, Sorting Intolerant From Tolerant (prediction software); SRNS, steroid-resistant nephrotic syndrome; Xt, *Xenopus tropicalis*.

Via GeneMatcher,^35^ we identified four additional variants in *DLG5* in patients (II 1–1, II 2–1, III 2–1 and IV 2–1) from three unrelated families with congenital abnormalities. Patients II 1–1 (father) and II 2–1 (son) are both heterozygous for a novel variant c.4526_4527delinsTT, p.Gln1509Leu (figure 1E). The father has right uteropelvic junction (UPJ) obstruction as well as naevus pigmentosus on the abdomen. The son also has UPJ obstruction, bilaterally and naevus pigmentosus on the abdomen. In addition, the son had hydronephrosis (figure 1C) and hydroceles along with other congenital abnormalities including mild facial dysmorphism (narrow, high arched palate, slightly bent right auricle) and increased distance between the hallux and the second digit of the foot. Neither of these patients had signs of hydrocephaly or CHD.

Patient III 2–1 is compound heterozygous for the variants c.497G>A, p.Arg166His and c.3214C>T, p.Arg1072Cys (figure 1E), which have respective frequencies of 2.03e-5 and 5.56e-4 with no homozygous individuals in the gnomAD database (table 1). She presented at 7 years of age with kidney dysfunction in the form of SRNS, with accompanying oedema, ascites, pleural effusions, hypertension, proteinuria (7.5 g/day), a serum creatine level of 0.8 mg/dL and a serum albumin of 20 g/L. The patient was treated with cyclosporin A but has had several relapses and has progressed to stage 3 chronic kidney disease. This patient showed no signs of extrarenal disease, although the patient had an isolated history of seizures and coma, symptoms attributed to a hypertensive crisis. A CT scan at that time showed slightly dilated cerebral ventricles.

Patient IV 2–1 was homozygous for the novel variant c.2461C>T, p.Arg821Ter. The patient was identified antenatally with last follow-up at 7 months of age. The patient was found to have communicating hydrocephalus, coarse facial features, a cleft lip and palate, broad hands/feet, contractures of the hands, short trunk/chest and intermittent dystonia. The patient failed to achieve developmental milestones. The patient had a normal echocardiogram. Although renal scans were normal, the patient was known to have renal tubular leak and salt wasting requiring administration of supplements.

None of these variants have previously been associated with human disease.

### Depletion of *Xenopus dlg5* disrupts kidney and brain ventricle morphology

Although we had little clinical evidence for a defect in cilia, previous work has elucidated a role for Dlg5 in cilia. *Dlg5* knockout mice have both hydrocephalus and cystic kidneys with a loss of cilia in both tissues.^22^ Another report has indicated that DLG5 is located at the base of cilia and regulates Shh signalling.^19^ Some *DLG5* variant probands presented with a constellation of phenotypes that are consistent with ciliopathies. Given the variability in phenotypic severity in *DLG5* variant patients, we hoped to establish a disease model in which we could probe disease mechanism and variant impact.

We first assessed *dlg5* expression in *Xenopus* by using WISH to determine patterns of expression. Regions corresponding to the brain, pronephros, heart and neural tube express *dlg5* (online supplementary figure 2). As these tissues are relevant to the pathologies observed in patients, we speculated that modelling *dlg5* dysfunction in *Xenopus* would be useful for understanding disease processes in these regions. We used morpholino oligo (MO) knockdown or F0 CRISPR knockout in *Xenopus* to test for cilia phenotypes and evaluate the pathogenicity of patient variants. *Xenopus* is ideal for this because generating embryos with specific gene depletion is time-efficient and cost-effective by simply microinjecting either MOs or sgRNA/Cas9 into the one cell embryo. Additionally, *Xenopus* lies at an ideal evolutionary balance between the high cost and low throughput of mammalian models and other rapid and inexpensive animal models.^36 37^ Another advantage of *Xenopus*, by microinjecting a two-cell stage embryo, we can analyse embryos in which one side of the embryo is depleted of *dlg5* and use the other side as an internal control.

**Figure 2:**
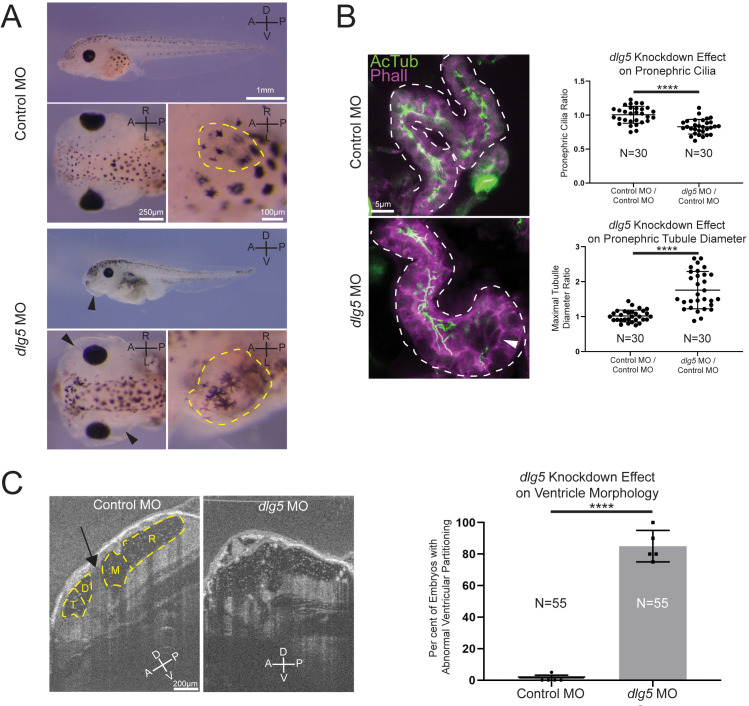
Knockdown of *discs large 5* (*dlg5)* in *Xenopus* embryos causes kidney and brain ventricle dysmorphology. (A) Representative images of stage 45 embryos injected with either a standard control MO or *dlg5* MO show oedema (black arrow head) and kidney dysplasia (outlined in yellow) resulting from *dlg5* knockdown. (B) Representative images of control MO and *dlg5* MO-injected sides of stage 45 embryos along with quantitation reveal a loss of cilia and increased proximal tubule diameter in the pronephroi. Each data point is the ratio within a single embryo of either cilia detected or maximal tubule diameter in the proximal tubule between two sides either both treated with control MO (Control MO/Control MO) or one treated with *dlg5* MO and the other treated with control MO (*dlg5* MO/Control MO). (C) Representative images and quantitation of stage 45 embryos injected with either a standard control MO or *dlg5* MO imaged via optical coherence tomography demonstrate the ventricular dysmorphology that results from *dlg5* knockdown. The arrow designates the typical division between ventricles that can be found lateral to the brain midline. Embryonic axes are labelled for reference. A, anterior; D, dorsal; P, posterior; V, ventral; L, left; R, right. Ventricles are labelled according to the adjacent subdivisions of the embryonic brain. T, telencephalon; D, diencephalon; M, mesencephalon; R, rhombencephalon. Statistical tests carried out as two-tailed t-tests with ****p<0.0001 (A) or an unpaired t-test with ****p<0.0001 (C). Bars indicate mean and SD of individual values (B) or replicate means (C).

Given that our patients had a variety of kidney abnormalities including UPJ obstruction, hydronephrosis, cystic kidneys and nephrotic syndrome, we examined the kidneys in our *Xenopus* model. Both MO knockdown and F0 CRISPR knockout of *dlg5* at the one-cell stage resulted in anasarca in tadpole stage embryos (figure 2A and online supplemental figure 3A). This is similar to previously described phenotypes related to cystic kidneys in *Xenopus*.^38 39^ During normal development of the pronephros, the amphibian embryonic kidney, cilia are present throughout the tubular structures of this organ.^40^ Depletion of *dlg5* led to an enlarged appearance of kidney tubules (figure 2A and online supplemental figure 3A, dashed yellow outlines in insets). On closer assessment of the proximal tubules of tadpole pronephroi in embryos depleted of *dlg5* on one side, there was a clear enlargement of tubules (figure 2B and online supplemental figure 3B). These regions in particular appeared to have fewer cilia (figure 2B and online supplemental figure 3B arrowheads).

**Figure 3:**
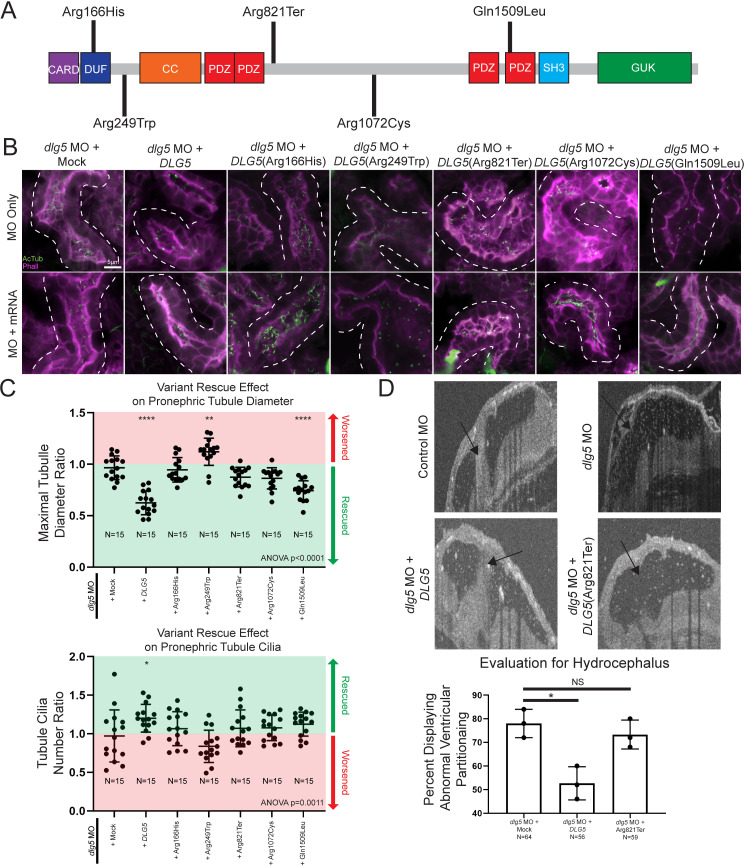
Xenopus model of discs large 5 (dlg5) loss of function provides a platform for testing variant pathogenicity. (A) Schematic diagram of DLG5 protein with domain annotations and locations and effects of uncovered patient variants. CARD, caspase activation and recruitment domain; DUF, domain of unknown function; CC, coiled-coil domain; PDZ, postsynaptic density protein disc large tumour suppressor and zonula occludens-1 domain; SH3, src homology 3 domain; GUK, membrane-associated guanylate kinase domain. (B) Representative images of proximal pronephric tubule ciliation and morphology. Dotted lines indicate bounds of tubule assessed in the image. (C) Quantitation of cilia and tubule morphology rescue efficiency of wildtype and variant DLG5 mRNA demonstrate the differing functionality of DLG5 variants. Ratios are calculated as values from sides with mRNA along with MO divided by values from MO only sides. Red indicates ratios suggesting an exacerbation of the phenotype while green indicates ratios suggesting rescue of the phenotype. (D) Representative images and quantitation of brain ventricle morphology rescue efficiency of wildtype and p.Arg821Ter variant DLG5 mRNA demonstrate the loss of functionality of the p.Arg821Ter DLG5 variant. Statistical tests carried out as analysis of variance (ANOVA) followed by Dunnett’s multiple comparison test with ****p<0.0001, **p<0.005 and *p<0.05 (C) or an unpaired t-test test with *p<0.05 (D). Bars indicate mean and SD of individual values (C) or replicate means (D).

To address the specificity of our depletion strategies, we detected similar phenotypes when we depleted *dlg5* either by MO or CRISPR that was not detected when compared with control MO or Cas9 only injections. In addition, we could rescue the MO phenotypes with the reference human mRNA but not some of the patient variants (figure 3). Finally, we were able to verify correct genomic CRISPR/Cas9 editing via tracking of indels by decomposition (online supplemental figure 3C). This approach allows us to quickly assay, characterise and quantify mutations induced by CRISPR/Cas9 and revealed a predominance of out-of-frame insertions and deletions that are likely to disrupt gene function.^41^ From these data, we conclude that Dlg5 is necessary for proper ciliation and development of the kidney.

Given the presentation of one of our probands with hydrocephalus, we also examined the brains of morphant and crispant embryos and assessed the cerebral ventricles using OCT. This imaging modality is analogous to ultrasound but uses light rather than sound to generate an image. In *Xenopus*, we can visualise the entire ventricular system and detect flow within and across ventricle spaces due to endogenous particles that conveniently provide the necessary contrast.^32^ First, we compared the ventricle spaces between control embryos and *dlg5* morphants (figure 2C). We observed a marked loss of midline structures analogous to the human cerebral aqueduct and foramen of Monroe consistent with a phenotype of communicating hydrocephalus (figure 2C, arrow indicates aqueduct). Within the ventricular system, beating cilia generate fluid flow. Interestingly, flow within the cerebral ventricles persisted even in this abnormal morphology suggesting some residual cilia function or prevailing choroid plexus-driven flow (online supplementary movie S1-S2).

10.1136/jmedgenet-2019-106805.supp1 Supplementary video

10.1136/jmedgenet-2019-106805.supp2 Supplementary video

From these results, we conclude that DLG5 affects kidney and brain ventricle development. In addition, kidney cilia are also compromised in our animal model.

### *dlg5* loss results in polarity defects and disruption of Shh signalling

**Figure 4:**
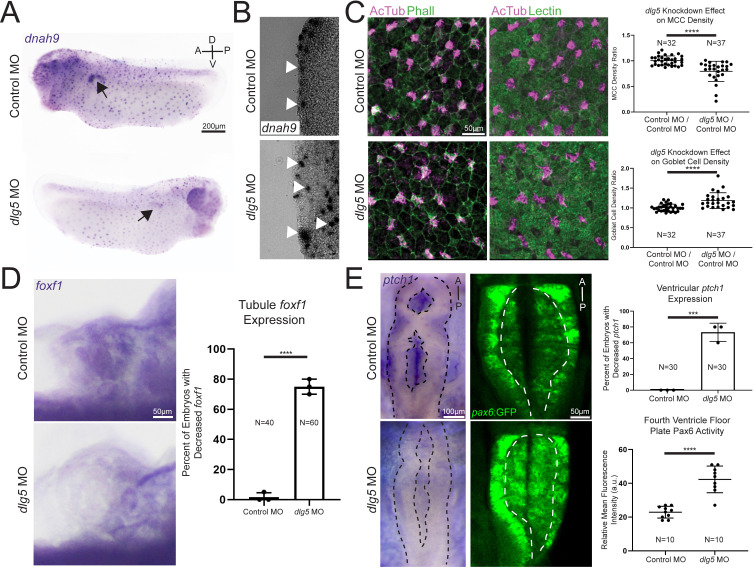
Knockdown of *discs large 5* (*dlg5)* in *Xenopus* embryos causes cell polarity and Hedgehog signalling dysfunction. (A) Representative images of *dnah9 in situ* hybridisation in control MO and *dlg5* MO sides of a stage 28 embryo depict fainter expression signal in the *dlg5* depleted side. (B) Representative transverse sections of control MO and *dlg5* MO sides of a stage 28 embryo reveal the more basal location of cells expressing the *dnah9* marker of MCC cell fate. (C) Representative immunofluorescence images and quantitation of AcTub-positive ciliated cells/area and Lectin-positive goblet cells/area in control MO and *dlg5* MO sides of a stage 28 embryo demonstrate the decreased density of MCCs and concomitant increased density of goblet cells in *dlg5* depleted *Xenopus* epidermis. (D) Representative WISH for *foxf1* Hedgehog target expression in control MO and *dlg5* MO stage 45 pronephroi demonstrate the decreased Hedgehog signalling activity in *dlg5* depleted pronephroi. (E) Representative expression and quantitation of *ptch1* in control MO and *dlg5* MO stage 45 brain ventricles as well as representative expression and quantitation of Pax6 in control MO and *dlg5* MO stage 45 fourth ventricle demonstrate a decrease in *ptch1* expression and expansion of Pax6. Black dotted line delineates bounds of the brain and white dotted line delineates bounds of fourth ventricle floor. A, anterior; D, dorsal; P, posterior; V, ventral. Statistical tests carried out as two-tailed t-tests for cell density (C) and Pax6 expression (E) and unpaired t-tests for *foxf1* (D) and *ptch1* (E) expression with ***p<0.005 and ****p<0.0001. Bars indicate mean and SD of individual values (E—Pax6 expression) or replicate means (D and E*—foxf1* or *ptch1* expression).

Because some probands had ciliopathy phenotypes and we detected a loss of cilia in the *Xenopus* pronephroi, we further characterised cilia in additional *Xenopus* tissues. Similar to other multiciliated epithelia such as the mammalian respiratory tract or oviduct, the *Xenopus* embryonic epidermis has an array of multiciliated cells that beat vigorously creating a brisk bulk fluid flow over the surface of the embryo.^42 43^ First, we examined early multiciliated cell fate specification by WISH) for *dnah9*, which is a marker of ciliated cell fate. This revealed similar numbers of labelled cells between *dlg5* MO-injected sides and control. However, we noticed that cell staining of *dnah9* expression appeared consistently weaker on the side injected with *dlg5* MO (figure 4A). Normally, multiciliated cells originate at the basal layer of the epidermis and then migrate apically to insert between epithelial cells so that the cilia have access to the extraembryonic fluid.^44–46^ Cross-sectional analysis of the *dlg5* morphants revealed that the cells labelled for ciliated cell fate were not at the epidermal surface as in the control, but were rather clustered beneath this layer (figure 4B), suggesting a failure of apical migration. To confirm this suspicion, we directly assessed cilia present in the embryonic epithelium. Depletion of *dlg5* in *Xenopus* embryos resulted in a loss of cilia from the surface of multiciliated epidermal cells (figure 4C). Consistent with a loss of multiciliated cells from the epidermal surface, there was a concomitant increase in density of secretory cells (figure 4C).

A previous report indicated that DLG5 is located at the bases of cilia and regulates Shh signalling, which supports a role in cilia function.^19^ We thus examined this pathway in the context of *dlg5* depletion. Within the developing mesoderm, Foxf1 is a well-established Shh target gene and is a critical transcription factor for proper development of mesoderm-derived organs such as the pronephros.^47 48^ In addition, this marker seemed particularly relevant as *FOXF1* dysfunction itself can give rise to CAKUT pathology.^49 50^ We thus used the expression of this marker as a readout of Shh signalling in the pronephros and observed a decrease in *foxf1* expression levels in pronephroi subjected to *dlg5* depletion via WISH (figure 4D). Within the developing brain, Shh, emanating predominantly from the underlying notochord, patterns the tissue in a stereotyped ventral to dorsal axis.^51 52^ As a direct target of Shh, *ptch1* expression in the neural tube and brain mirrors the distribution and activity of Shh and can serve as a readout of this pathway.^53 54^ We therefore used *ptch1* to monitor Shh signalling in the brain ventricles of control and *dlg5* morphant embryos and observed a stark reduction of this marker in embryos depleted of *dlg5* (figure 4E). Pax6 is normally expressed in the lateral margin of the neural tube due to repression by Shh signalling ventrally, and therefore loss of Shh should lead to expansion of *pax6* expression ventrally towards and including the ventricular floor later in development. To test this, we again employed knockdown in *Xenopus* to analyse this pattern on *dlg5* depletion. In a transgenic *Xenopus* line where GFP expression is driven by a *pax6* promoter, we observed an increase in the domain of Pax6 expression in the ventricular floor (figure 4E).

### Cilia deficits are apparent in a subset of proband tissues

**Figure 5:**
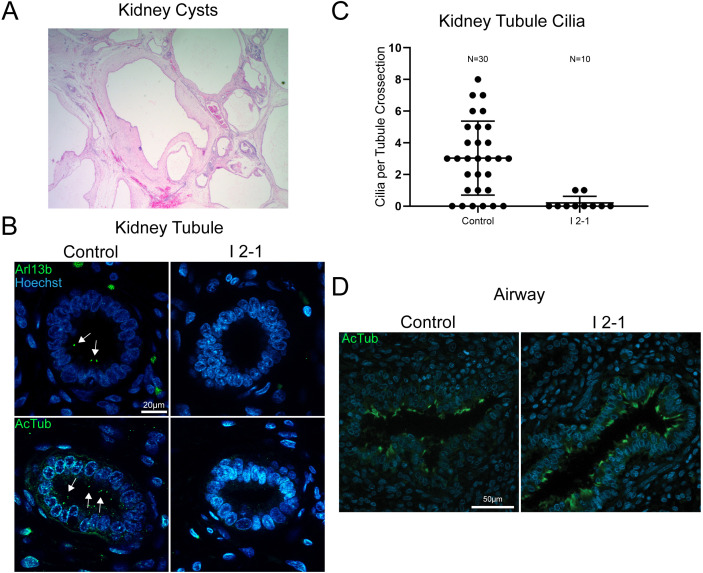
Proband tissue bearing *discs large 5* (*DLG5)* variant c.745C>T (p.Arg249Trp) displays tissue-specific deficits in ciliation. (A) Representative H&E stain of I 2–1 kidney showing cystic morphology. (B) Control 23 weeks and I 2–1 weeks fetal kidney tissue cross-section immunofluorescence labelling of cilia Arl13b or acetylated tubulin show a loss of cilia in DLG5 variant tissue. (C) Quantitation of cilia number in each cross-sectional kidney tubule analysed. (D) Control 23 weeks and I 2–1 weeks fetal lung airway tissue cross-section immunofluorescence labelling of cilia acetylated tubulin shows intact airway ciliation in *DLG5* variant tissue. Bars indicate mean and SD of individual counts.

Since we observed cilia deficits in *Xenopus* embryos depleted of *dlg5*, we postulated that cilia defects might also be present in tissues obtained from the proband (I (2–1)). Kidney tissues were highly cystic (figure 5A), and we detected far fewer cilia compared with a stage matched control using antibodies against α-acetylated tubulin or ARL13b, a regulatory GTPase that localises to primary cilia (figure 5B C).^55 56^ In order to determine how generalised the cilia loss might be, we sought to assess additional, available ciliated tissues such as the multiciliated cells of the airway. In contrast to renal cilia, airway cilia in the proband appeared completely intact when compared with the stage matched control tissue (figure 5D). In summary, DLG5 dysfunction seems to affect some but not all ciliated tissues. This may contribute to the tissue proclivity seen across our patients.

### Patient *DLG5* variants are dysfunctional in *Xenopus*

In addition to the tissue specific effects of DLG5, we noted that patients had different inheritance patterns (dominant or recessive) along with remarkable variability in severity of phenotypes. Perhaps most notable, proband I 2–1 had a de novo p.Arg249Trp mutation and severe cystic kidneys compared with II 1–1 or 2–1 who were heterozygous for a p.Gln1509Leu variant, but had relatively mild UPJ obstruction with or without hydronephrosis. Therefore, we sought to investigate the impact of these variants on DLG5 function in *Xenopus* in order to better understand this phenotypic variability (figure 3A). Since multiple patients had kidney phenotypes, we focused on kidney tubule cilia and morphology and used a rescue strategy to evaluate the impact of patient variants. We depleted *dlg5* in the entire *Xenopus* embryo by injecting *dlg5* MO at the one cell stage, and then injected mRNA for the wildtype human *DLG5* or the patient variants into one cell at the two-cell stage. In this way, we compared tubule cilia and morphology between the two sides of the embryo to evaluate the degree of rescue, if any. Wildtype *DLG5* mRNA ameliorated the defects in both cilia number and morphology indicating that our MO is specific to *dlg5* (figure 3B, C). Introduction of mRNA derived from three of the variants associated with abnormal kidney morphology and/or function (p.Arg166His, p.Arg249Trp, and p.Arg1072Cys) did not significantly improve pronephric appearance (figure 3B, C). In fact, the p.Arg249Trp variant seemed to significantly exacerbate the dysmorphology found in tubules. Interestingly, although the respective patient had only minor functional kidney disease, we did test the p.Arg821Ter variant, which also failed to rescue pronephric morphology (figure 3B, C). Only variant p.Gln1509Leu resulted in reestablishment of cilia number and tubule morphology (figure 3B, C). Based on the phenotype exacerbation with variant p.Arg249Trp, we sought to determine whether overexpression of this variant was sufficient to cause dysmorphology. Indeed, introducing this variant in wildtype embryos appeared to cause dilation of the pronephros similar to knockdown of *dlg5* (online supplementary figure 4).

Next, we sought to examine the hydrocephalus phenotype. In this case, we co-injected both the *dlg5* MO along with either wildtype or patient variant *DLG5* mRNA and then examined the ventriculomegaly phenotype in the embryonic brain. The wildtype *DLG5* mRNA did indeed improve the morphology of the ventricles comparing *dlg5* morphants and control embryos. Consistent with a loss-of-function allele, the *DLG5* sequence variant identified in a proband with hydrocephalus (p.Arg821Ter) did not significantly improve the ventricular size caused by Dlg5 depletion. From these results, we conclude that *dlg5* is critical for multiple phenotypes in the embryo including hydrocephalus and kidney abnormalities, likely due to a functional role for Dlg5 in ciliated cells.

## Discussion

We have described a constellation of phenotypes in probands with variants in *DLG5* displaying a variety of modes of inheritance and zygosity. In our search for an explanation for these phenotypes, we discovered ciliary defects associated with the knockdown of *dlg5* in our *Xenopus* model, as well as in the tissue from our proband. Multiple lines of evidence suggest polarity dysfunction and a subsequent loss of cilia in the context of *DLG5* loss of function. Our *Xenopus* model demonstrated various phenotypes—including enlarged kidney tubules, loss of kidney cilia and hydrocephalus—that were consistent with cilia dysfunction and reflected features found in our patients. Turning then to tissue obtained from the original proband that led to this study, we discovered that cilia were also absent from the remaining tubular structures of largely cystic kidneys while those within the airway tissue were present, pointing to potential tissue specificity for damaging effects of *DLG5* variants.

A knockout mouse model of Dlg5 greatly contributed to our understanding of potential relationships between Dlg5 interactants and disease mechanisms.^22 23^ The Dlg5 mouse knockout model displayed cystic kidneys and hydrocephalus as well as loss of cilia. These abnormalities were attributed to improper establishment of cell polarity. In support of this role, DLG5 may create a scaffold between vinexin-vinculin and β-catenin at cell-cell junctions.^24^ Cell-cell junction maintenance is crucial for cell polarity.^57^ Dysfunctional cell polarity has previously been identified as a mechanism for podocyte dysfunction and subsequent nephrotic syndrome pathogenesis.^58^

With regard to cilia phenotypes, ciliated cells are inherently polarised as the cilia must extend from the apical side of the cell. Interestingly, in the case of the multiciliated cells of the *Xenopus* embryonic epidermis, these cells are specified in the deep layers and migrate in a polarised fashion apically so cilia can gain access to the extraembryonic fluid. In this context, depletion of *dlg*5 leads to the multiciliated cells remaining in the deep layers suggesting that they cannot undergo these polarised behaviours.

In both mouse and *Xenopus*, loss of DLG5 leads to hydrocephaly; however, at least in *Xenopus*, ventricular flow appears somewhat intact based on OCT imaging. Therefore, the hydrocephalus may be due to abnormal signalling downstream of cilia flow, or conversely may be due to mis-patterning of the brain.^59 60^ In this latter regard, DLG5 at the cilium appears to modulate Shh signalling via interactions with Smo.^19^ Shh signalling in turn orchestrates embryonic patterning including that of the digits of the limb and the kidney.^61–63^ Indeed, Shh signalling appeared disrupted based on the decreased expression of *foxf1* and the expansion of Pax6 expression observed on *dlg5* depletion. Of note, DLG5 has many functions including modulation of Hippo and TGF-β signalling, so future studies will need to determine which of these pathways impacts different tissues with loss of DLG5 function in patients.^20 21 24 64^

Clinically, genotype/phenotype relationships are only now emerging for *DLG5*, which given the wide-ranging variant types and phenotypes, demands that we improve our rudimentary understanding in order to better appreciate disease pathogenesis in our patients. Recently, a brief report described a patient whose phenotype encompassed a number of the congenital findings we witnessed in individuals in our cohort, including hydrocephalus, CHD (atrial and ventricular septal defects), cleft lip and palate and cystic kidney disease. This patient, the product of a consanguineous union, had a homozygous frameshift variant in *DLG5* (c.3081_3106del26, p.Arg1027Argfs10*).^65^ In addition, the healthy parents have a history of an intrauterine fetal demise associated with ventriculomegaly and echogenic kidneys.

In our cohort of patients with DLG5 variants, it appears that disease severity may well follow different types and degrees of disruption in the protein. Like the patient with the homozygous frameshift variant mentioned above, patient IV 2–1 also displayed hydrocephalus along with craniofacial defects and mild limb abnormalities as a result of a homozygous truncating *DLG5* allele (p.Arg821Ter). Patients with homozygous variants p.Arg821Ter and p.Arg1027Argfs10* both have asymptomatic carrier parents. Additionally suggesting recessive inheritance, patient III 2–1 had compound heterozygous missense variants p.Arg166His and p.Arg1072Cys, which appeared to be loss of function for DLG5 in our frog model and were each carried by healthy parents.

However, dominant variants are also described in our cohort. The *de novo* variant (p.Arg249Trp) identified in the I 2–1 fetus with CAKUT, CHD and limb abnormalities appears to have severe phenotypic consequences despite arising as a single allele. In contrast, the patients II 1–1 and II 2–1 with the p.Gln1509Leu allele have a milder CAKUT phenotype. Our functional studies of patient variants give some insight to these observed differences in phenotype. Interestingly, the p.Arg249Trp allele appears to have a “dominant negative” effect. Rescue experiments with p.Arg249Trp exacerbate the *dlg5* loss-of-function phenotype and overexpression of p.Arg249Trp itself leads to loss of cilia and dilation of renal tubules. This dominant negative variant is identified in an individual (I 2–1), who presented with a severe multisystem syndrome. In contrast, the p.Gln1509Leu variant has some degree of rescue in our *Xenopus* assay, although not as significant as the wildtype rescue suggesting that it may be a hypomorphic allele. This p.Gln1509Leu variant is identified in family II who are more mildly affected than individual I 2–1. Why the son II 2–1 is more impacted than his father II 1–1 remains to be explained. Intrafamilial and interfamilial differences may be due to inherent variable expressivity of *DLG5* phenotypes or may be the influence of other unknown factors.

A number of studies have interrogated several of the DLG5 domains necessary for interactions with other proteins that have well-established functions in a variety of cell biological roles and signalling pathways.^19–22 24^ Using knockdown coupled with replacement with wildtype or variant DLG5 in *Xenopus* embryos, we provide evidence that the DLG5 alleles identified in our study impair protein function. The established DLG5 interaction map (online supplementary figure 5) allows us to make predictions about the impact of these variants on crucial interactions with other proteins, although further interrogation of these interactions is necessary to understand their role in disease.

The phenotypic results of DLG5 variants may be second to fundamentally different impairments of function, related to variant type as well as location within the DLG5 protein and subsequently altered interactions with other proteins. Nevertheless, while difficult to establish firmly given our small sample size, *DLG5*-related disease appears to be variably inherited and ranges clinically from mild to severe with neurological, renal, craniofacial, cardiac and limb defects. Additional patients and research are needed to understand the full extent of congenital defects associated with variants in this gene, and what genetic, environmental or other modifying influences would further explain specific observable differences.

## Data Availability

Data are available on reasonable request. All data relevant to the study are included in the article or uploaded as supplementary information.
